# The Myokine FGF-21 Responds in a Time-Dependent Manner to Three Different Types of Acute Exercise

**DOI:** 10.3390/muscles5010003

**Published:** 2026-01-04

**Authors:** Mikal Thrones, Thomas Rawliuk, Dean M. Cordingley, Stephen M. Cornish

**Affiliations:** 1 Faculty of Kinesiology and Recreation Management, University of Manitoba, Winnipeg, MB R3T 2N2, Canada; mikal.thrones@umanitoba.ca (M.T.); umcordid@myumanitoba.ca (D.M.C.); 2 Brain and Cognitive Sciences, Department of Psychology, University of Manitoba, Winnipeg, MB R3T 2N2, Canada; rawliukt@myumanitoba.ca; 3 Applied Health Sciences, University of Manitoba, Winnipeg, MB R3T 2N2, Canada; 4 Pan Am Clinic Foundation, 75 Poseidon Bay, Winnipeg, MB R3M 3E4, Canada; 5 Centre for Aging, University of Manitoba, Winnipeg, MB R3T 2N2, Canada

**Keywords:** myokine, cytokine, high-intensity exercise, moderate-intensity exercise, resistance exercise, skeletal muscle, apelin, FGF-21, IL-6, IL-15, irisin

## Abstract

**Background**: The myokine response to various types of exercise may differ and influence the adaptations to various physiological systems in response to training. This study aimed to compare systemic myokines’ (apelin, interleukin-6 [IL-6], interleu-kin-15 [IL-15], fibroblast-growth factor-21 [FGF-21], and irisin) responses to acute moderate-intensity cardiovascular exercise (MICE), high-intensity interval exercise (HIIE), or resistance exercise (RE). **Methods**: Six healthy, recreationally active adults (n = 4 males, n = 2 females) completed this crossover pilot study. After baseline testing, in a balanced randomized order, participants completed all three exercise sessions with one week between each of the exercise sessions. Blood samples were obtained at rest, immediately post-exercise, and 1 and 3 h post-exercise. Myokine response was analyzed using a 3 (exercise condition: MICE, HIIE, RE) × 4 (time: baseline, post-exercise, 1 and 3 h post-exercise) repeated-measures ANOVA. **Results**: Our results showed no significant interaction of time × exercise type in any of the analyzed myokines (all *p* > 0.05). A significant main effect of time was found for FGF-21, where concentrations at baseline (188.96 ± 127.34 pg/mL; *p* = 0.038) and immediately post-exercise (206.27 ± 135.95 pg/mL; *p* = 0.006) were higher than 3 h post-exercise (111.08 ± 127.65 pg/mL). No other main effects for time or exercise type were identified (all *p* > 0.05). **Conclusions**: The three exercise types, when analyzed together in this study, demonstrated a reduction in FGF-21 3 h post-exercise, suggesting this myokine was removed from the systemic circulation following exercise. The negative results of this study are inconclusive given the lower statistical power observed in this research. These preliminary results indicate the need for a larger trial to evaluate the effects of different types of exercise on the specificity of myokine responses and how acute exercise responses may translate into long-term exercise training adaptations.

## 1. Introduction

Myokines are defined as cytokines or proteins/peptides that are released from skeletal muscle tissue when it is contracted forcefully or for extended periods of time such as during exercise. Myokine research has recently gained a large degree of attention in the exercise science literature [1]. For example, myokines are thought to be involved in hypertrophy of skeletal muscle, glucose and lipid metabolism, “browning” of white adipose tissue, maintenance or development of bone tissue, and reductions in visceral fat mass [2,3,4,5,6,7,8]. Specifically, there are many different types of myokines that are involved in a variety of biological processes in relation to exercise; however, there has been minimal research investigating how different types or modalities of exercise influence the systemic myokine response in humans.

Interestingly, apelin has been found to increase systemically with resistance exercise in humans [4] and has shown an ability to reverse sarcopenia in older mice injected with a recombinant form [3] by up-regulating a pathway of anabolism in skeletal muscle. FGF-21 has known effects on both glucose and lipid metabolism [7]; active individuals have lower resting FGF-21 concentrations than sedentary individuals [6]. Studies on IL-6 demonstrate this myokine plays multiple roles in relation to glucose and lipid metabolism [2], skeletal muscle hypertrophy [9], and the anti-inflammatory effects of exercise training [10]. IL-15 has a role in both skeletal muscle anabolism [11], as well as in reductions in visceral ‘pro-inflammatory’ fat deposits [11]. Irisin has been shown to increase with acute exercise and may be involved in the ‘browning’ of white adipose tissue [12] which is known to increase the energy expenditure capabilities of adipose by up-regulating uncoupling proteins [12].

Unfortunately, there is limited research on which types of exercise best produce a myokine response. Previous research in this area [2,3,5,8,11] has evaluated the systemic response of a variety of myokines to individual exercise stressors; however, there are very few research studies [13,14,15] that have looked at differences between various types of exercise and the acute myokine response within a single study design. One research study identified the differences and similarities in a variety of myokines and their response to high-intensity interval training (HIIT) and resistance exercise (RE) acutely [15]. In this study, RE was able to increase FGF-21 more so than HIIT, and HIIT was able to increase follistatin more than RE [15]. Thus, it appears that differences exist between the type of exercise and the expected myokine response. Of note, this study also evaluated the myokines IL-15, irisin, and myostatin, which all had a similar response to the HIIT or RE protocols. We have recently evaluated different loading patterns and different types of RE and found that there were no differences in IL-6, IL-15, or decorin response when altering these parameters associated with an acute bout of RE. To extend this previous work, we evaluated three types of exercise (moderate-intensity continuous exercise [MICE], high-intensity interval exercise [HIIE], and RE) to determine if differences or similarities exist between these types of popular exercise modalities and the systemic myokine response associated with them. Thus, the purpose of this research is to determine if there are differences between three different types of acute exercise in relation to systemic myokine (apelin, FGF-21, IL-6, IL-15, and irisin) responses. The main hypotheses of this research study are that (1) apelin and IL-15 will respond to a higher degree with RE when compared to MICE or HIIE and (2) FGF-21, IL-6, and irisin will respond to a higher degree with MICE and HIIE when compared to RE. The rationale for the suspected alteration in responses between different types of exercise is due to the differing acute perturbations in molecular pathways that are observed with different types of exercise, and which metabolic system pathways are most affected by the type of exercise.

## 2. Results

**Figure 1:**
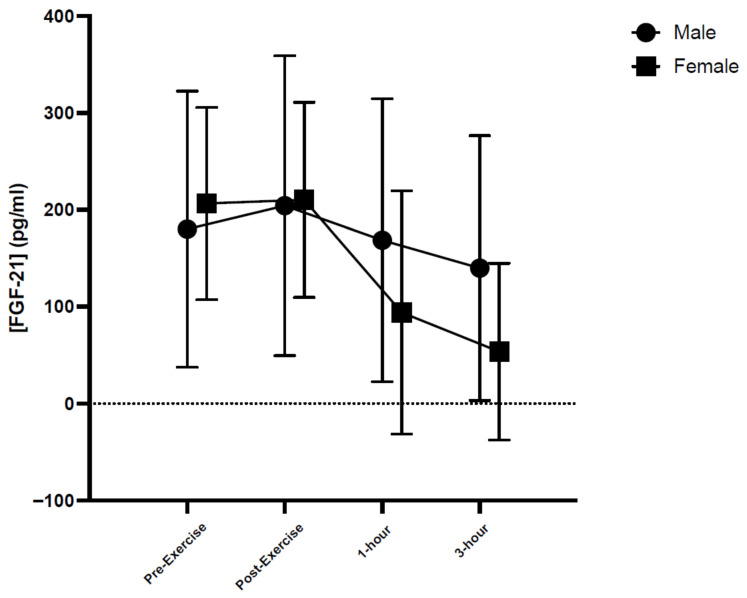
Sex × time based means ± standard deviations (SD) for FGF-21 concentrations.

The participants’ baseline characteristics are shown in Table 1. Mauchly’s test of sphericity was non-significant in all cases for the myokines (*p* > 0.05); however, IL-15 data did not meet the assumption of sphericity (*p* = 0.0002). In this instance Greenhouse–Geiser correction was not applied as the interaction and main effects were non-significant. There were no significant interaction effects for time × exercise type for any of the measured myokines (apelin: F(6, 45) = 0.741, *p* = 0.619, η_p_^2^ = 0.090; FGF-21: F(6, 45) = 1.011, *p* = 0.430, η_p_^2^ = 0.119; irisin: F(6, 45) = 2.004, *p* = 0.085, η_p_^2^ = 0.211; IL-6: F(6, 45) = 0.183, *p* = 0.980, η_p_^2^ = 0.024; IL-15: F(6, 45) = 0.997, *p* = 0.439, η_p_^2^ = 0.117; see Table 2). There was a main effect of time for FGF-21: F(3, 45) = 5.047, *p* = 0.004, η_p_^2^ = 0.252, where post hoc analysis indicated that baseline (*p* = 0.038) and immediately post-exercise (*p* = 0.006) were significantly higher than 3 h post-exercise (see Figure 1 for sex × time analysis); however, no other main effects of time were noted for the other myokines of interest in this study (apelin: F(3, 45) = 1.023, *p* = 0.391, η_p_^2^ = 0.064; irisin: F(3, 45) = 0.717, *p* = 0.547, η_p_^2^ = 0.046; IL-6: F(3, 45) = 1.385, *p* = 0.260, η_p_^2^ = 0.085; IL-15: F(3, 45) = 0.793, *p* = 0.504, η_p_^2^ = 0.050; see Table 2). Further, there were no main effects of exercise type on the myokine response in this study (apelin: F(2, 15) = 0.389, *p* = 0.684, η_p_^2^ = 0.049; FGF-21: F(2, 15) = 0.421, *p* = 0.664, η_p_^2^ = 0.053; irisin: F(2, 15) = 0.324, *p* = 0.728, η_p_^2^ = 0.041; IL-6: F(2, 15) = 0.421, *p* = 0.664, η_p_^2^ = 0.053; IL-15: F(2, 15) = 0.152, *p* = 0.860, η_p_^2^ = 0.020; see Table 2).

## 3. Discussion

The present pilot study investigated the acute systemic myokine response to three distinct exercise modalities in young, healthy, recreationally active adults. Contrary to our hypothesis, we found no significant interaction effects between exercise type and time for any of the five myokines examined. The most notable finding was a significant main effect of time for FGF-21, which showed higher concentrations at baseline and immediately post-exercise compared to 3 h post-exercise, independent of exercise modality. These findings suggest that acute exercise-induced myokine responses may be less exercise-specific than previously anticipated, at least within the parameters and population examined in this pilot investigation.

FGF-21 concentrations were significantly higher at baseline and immediately post-exercise compared to 3 h post-exercise, regardless of exercise type. FGF-21 is recognized as a key metabolic hormone involved in glucose homeostasis, lipid metabolism, and energy expenditure regulation [7,8,16]. The observed decline in FGF-21 concentrations from baseline to 3 h post-exercise may reflect the mobilization of this myokine to areas of need following acute stress from exercise, or FGF-21 may be proteolyzed in circulation or cleared by the kidneys. Interestingly, the lack of exercise-specific differences in FGF-21 response suggests that the metabolic stress imposed by MICE, HIIE, and RE may be comparable in terms of FGF-21 regulation, at least within the acute phase of exercise and recovery. This finding contrasts with previous research which reported exercise modality-specific FGF-21 responses, where RE was able to increase the area under the curve for this same myokine to a greater degree than short-duration HIIE [15]. However, direct comparisons are challenging due to differences in study populations, sample sizes, exercise protocols, and sampling timepoints. We postulate that FGF-21 was moving from systemic circulation to different tissue types following the exercise sessions to produce its positive effects on metabolism. FGF-21 is a potent endocrine myokine and hepatokine that has effects on glucose and lipid metabolism as well as influences on insulin sensitivity in skeletal muscle [17]. It also acts as a stimulator for AMPK and thus affects many metabolic pathways to aid in fat mass management and weight loss by increasing energy expenditure [18]. A recent systematic review of FGF-21 responses indicated that this biomolecule has effects on energy expenditure through improved glucose uptake and lipid oxidation, as well as lowering inflammation and the “browning” of white adipose tissue [19]. Interestingly, a meta-analysis found that resting concentrations of FGF-21 increased after various types of exercise training (i.e., concurrent, aerobic, resistance); however, there was variability between the analyzed studies with an overall large Hedge’s g (1.58) effect size, but this meta-analysis was performed in those with established type II diabetes [20]. It may be that young healthy research participants do not systemically respond, at least acutely, with FGF-21 as readily as those research participants in the aforementioned study, who all had a metabolic condition that influences myokine response to exercise. Overall, the novel findings of our study and other research findings point to an endocrine role for a type of myokine that has known powerful effects on metabolic pathways. Further research into the clinical application of this myokine/hepatokine is essential to understand its role when it binds to the FGF receptor as well as its coreceptor β-klotho [19].

The absence of significant interaction effects for the measured myokines was somewhat unexpected, given the distinct physiological demands associated with MICE, HIIE, and RE. One explanation may be the small sample size, as the pilot nature of this study with only six participants limited statistical power to detect statistically significant differences. Myokine responses are known to vary considerably between individuals [4], and although the observed interaction effect sizes ranged from small to large (η_p_^2^ = 0.024–0.211), the study was likely underpowered to confirm these as statistically significant. Another consideration is the timing of blood sampling. The chosen timepoints may not have aligned with the peak concentrations of some myokines, which can display distinct kinetic profiles. For example, irisin may respond shortly after the onset of exercise and begin decreasing soon on cessation of the exercise activity [13,14]. More frequent or extended sampling may therefore be necessary to fully capture exercise-specific differences.

The absence of significant IL-6 responses was somewhat unexpected, given its well-established role as an exercise-responsive myokine. IL-6 is typically elevated following both aerobic and resistance exercise, with responses often linked to exercise duration, intensity, and total muscle mass involved [4]. In this study, the lack of detectable changes may be related to the relatively short duration of the exercise protocols or the small sample size and high variability in the myokine responses to the exercise interventions. In a previous meta-analysis of patients with type II diabetes who completed various forms of exercise training, IL-6 was reduced in the systemic circulation under resting conditions, indicating a potential reduction in chronic low-grade inflammation [20]. However, IL-6 is a pleiotropic myokine, usually increasing acutely after a bout of exercise, and plays an essential role in up-regulating IL-10 and IL-1ra which are anti-inflammatory in nature [4,10]. IL-6 also acts to stimulate the release of fatty acids and glucose from adipose tissue and glycogen stores in the liver, respectively, when acute exercise intensity or duration reduces glycogen stores intramuscularly [4]. Our current study may not have been of sufficient exercise intensity or duration to reduce intramuscular glycogen stores to an appreciable amount that would have resulted in the systemic release of IL-6 [21].

The lack of significant irisin responses also stands in contrast to previous reports of acute increases following various exercise modalities [22]. The absence of changes in this study may suggest that more intense exercise or a different blood sampling timeframe is required to elicit measurable systemic responses [13,14]. Alternatively, variability in assay sensitivity or sample size may have influenced the ability to detect subtle changes. There has been much debate on the effects that acute and chronic exercise have on irisin concentrations systemically; however, a recent systematic review and meta-analysis demonstrated that in overweight and obese individuals, irisin was systemically increased with exercise training (minimum of 8 weeks) and this increase was associated with a reduced body fat mass, improved thermogenesis, and an alteration in adipose phenotype (i.e., browning of white adipose tissue) [23]. Our results indicated no acute response with irisin to three varying forms of exercise, but our exercise intensity and duration may not have been hard or long enough to stimulate irisin release systemically.

For apelin and IL-15, the absence of significant responses aligns with some evidence suggesting these myokines may not consistently respond to single exercise bouts [11,24]. Apelin, which plays a role in cardiovascular regulation and glucose metabolism [1,5], may be more responsive to repeated training stimuli rather than acute exercise. Similarly, IL-15, while important for skeletal muscle hypertrophy and metabolism [25], may show delayed or more modest changes that were not captured within the chosen sampling timeframe.

Several methodological factors may have influenced our findings and should be considered as limitations when interpreting the results. Firstly, future studies should seek to replicate these findings with larger samples to ensure that potential differences in myokine responses are being detected. A post hoc power analysis using apelin data from this study indicated that with our six participants in this study, we only achieved 34% statistical power, indicating a high likelihood of a type II statistical error being committed. Thus, in future research, the number of participants needs to be increased to 20 to obtain 80% statistical power to decrease the risk of a type II error. Further, there was high variability in the measurement of most of our myokines in this study, likely influencing the statistical power of the study to detect differences between conditions. This is especially highlighted by the high variability noted in our irisin concentrations, as noted in our results, and with the small sample sizes, this points to the fact that larger samples are necessary to draw appropriate conclusions. Additionally, our study population consisted of young, healthy, recreationally active individuals, who may have exhibited different myokine responses compared to sedentary, overweight/obese, or older populations. Regular physical activity can lead to adaptations that reduce the magnitude of acute exercise-induced inflammatory and metabolic responses [10]. Future studies should examine different populations, including sedentary individuals or those with metabolic dysfunction, who may display unique myokine responses. As research continues to unfold in this area, it will become increasingly necessary to prescribe exercise based on personalized health information to adequately address the health issues for a variety of individuals with various morbid and co-morbid diseases. Our study was also limited as we attempted to recruit participants during the COVID-19 pandemic, and we had extreme difficulty in accomplishing this. In relation to this, our attrition rate was very high (i.e., 45%), which has limited the conclusions we can draw given the small sample size analyzed. Furthermore, our study was limited as it did not provide a standardized meal prior to each of the exercise bouts. While we asked participants to repeat the same dietary eating habits on each day of exercise testing, this does not guarantee that the participants were able to eat the same nor does it prevent the possibility that participants could have been in a fasted state when completing the exercise bouts. Future research in this area should provide standardized meals prior to exercise for participants to reduce the chance of dietary influence on the myokine response. Finally, although participants were instructed to maintain consistent dietary intake across testing sessions, the potential influence of nutritional status on myokine responses cannot be entirely excluded. Factors such as carbohydrate availability, protein intake, and overall energy balance can affect exercise-induced myokine release [26]. Although difficult to completely control in human studies, more stringent dietary control or standardized pre-exercise meals may therefore be necessary to minimize nutritional confounding factors.

Similar myokine responses across exercise modalities suggest that, from a systemic myokine perspective, different exercise types may provide comparable acute metabolic stimuli. This finding supports the concept that various exercise modalities can contribute health benefits, potentially through common myokine-mediated pathways. For individuals seeking to optimize myokine responses for health benefits, the choice of exercise modality may be less critical than previously thought, at least for acute responses.

The significant temporal changes in FGF-21 suggest that this myokine may serve as a useful marker of metabolic recovery following exercise. The decline from baseline/immediate post-exercise to 3 h post-exercise may reflect a mobilization of the myokine to other tissues, where it may be required to aid in restoration of metabolic homeostasis and could potentially be used to monitor recovery status following exercise [7]. Future studies should employ larger sample sizes to improve statistical power and include extended sampling periods (24–48 h post-exercise) to capture delayed myokine responses. Additionally, more frequent sampling in the immediate post-exercise period may help identify rapid changes that were missed in our protocol.

Research should explore more diverse exercise protocols, including different intensities, durations, and modalities (e.g., eccentric exercise, plyometric training) to better characterize exercise-specific myokine responses. Additionally, examining dose–response relationships may help identify optimal exercise prescriptions for maximizing beneficial myokine responses. Studies should include diverse populations, including different age groups, fitness levels, and health statuses, to better understand how individual characteristics influence exercise-induced myokine responses. Special attention should be given to populations that may benefit most from myokine-mediated exercise effects, such as individuals with metabolic dysfunction or age-related muscle loss. Future research should incorporate mechanistic approaches, including muscle biopsy sampling, to examine the relationship between local muscle myokine production and systemic concentrations. Additionally, investigating the functional consequences of exercise-induced myokine changes through downstream signaling pathway analysis would provide valuable insights into their physiological relevance.

In conclusion, this pilot study provides limited insights into the acute systemic myokine response to different exercise modalities in young, healthy adults. While we did not observe exercise-specific differences in myokine responses, the significant temporal changes in FGF-21 suggest that this metabolic regulator follows distinct kinetic patterns following acute exercise. The absence of exercise-specific responses may reflect the need for larger sample sizes that emphasize the effects of various types of exercise on myokine responses systemically and how these myokine responses translate into physiological adaptations that improve health and performance outcomes. Further, different populations, such as those with metabolic conditions, overweight/obesity, and various inflammatory conditions should be assessed to determine the most effective types of exercise for these populations. Also, alternative exercise protocols, in addition to the ones assessed in the current study, require evaluation to detect if meaningful differences exist between exercise types in terms of myokine responses and how they influence skeletal muscle, as well as other physiological systems. These findings contribute to our understanding of exercise-induced myokine biology and highlight important methodological considerations for future research. The results suggest that, from an acute myokine response perspective, different exercise modalities may provide similar systemic metabolic stimuli, supporting the flexibility in exercise prescription for health benefits while emphasizing the need for continued research to optimize exercise interventions for specific myokine-mediated outcomes.

## 4. Methods

### 4.1. Research Design

**Figure 2:**
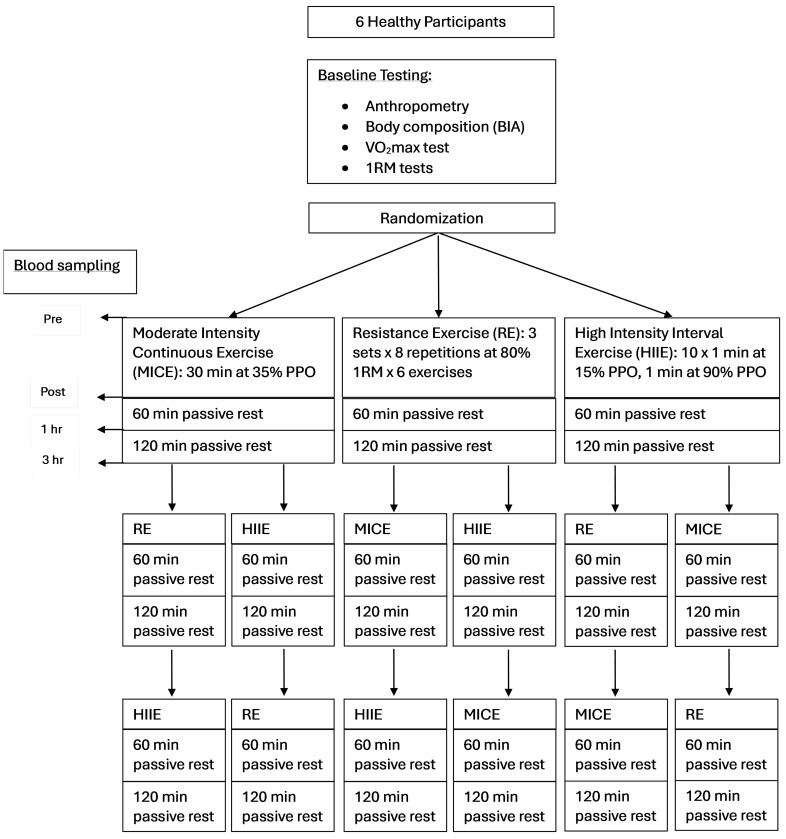
Schematic of study design for the crossover trial.

This research study was a repeated-measures crossover design, where participants acted as their own control, to evaluate the differences in systemic myokine response to three different exercise protocols in young healthy recreationally active adults. While many different modalities of exercise could have been evaluated, we evaluated three different exercise protocols that are popular amongst exercising individuals and included the following: (1) MICE; (2) HIIE; and (3) RE. The main dependent variables for this study were systemic blood-based myokines, which included apelin, FGF-21, IL-6, IL-15, and irisin. Participants had their blood sampled 4 times surrounding each exercise session (at rest, immediately after exercise, and 1 and 3 h post-exercise). A minimum of 1 week of rest between each exercise trial was given to allow for full recovery (see Figure 2). Furthermore, participants were asked to consume the same meals on the days surrounding their exercise sessions. At the first exercise session, participants were asked to record their dietary intake on a 3-day food log so this could be repeated on the days they participated in the two other types of exercise. The participants recorded their food intake on the two days before the exercise session and the day of their exercise session. Participants were also asked to avoid moderate to vigorous physical activity/exercise for 72 h prior to the first exercise session they participated in to avoid confounding the results.

Prior to completing the above three acute exercise conditions, each participant underwent standard laboratory testing to determine their (1) resting heart rate (bpm) and blood pressure (mmHg); (2) height (cm); (3) body mass (kg); and (4) fat mass and fat-free mass via bioelectrical impedance analysis (InBody 270 analyzer ©2015 InBody Co., Ltd., Cerritos, CA, USA). Further, $\dot{V}$O_2_peak (i.e., maximal oxygen consumption) was determined using a maximal cycle ergometer protocol (i.e., starting at 0.5 and 1.0 kiloponds [kp] for females and males, respectively, and increasing by 0.5 kp every 2 min until volitional exhaustion while maintaining a 60 rpm cadence) and a metabolic cart to assess oxygen consumption in each participant. The criteria for reaching $\dot{V}$O_2_peak was any three of the following: (1) ≥estimated maximal heart rate (220-age); (2) ≥17 rating of perceived exertion on the Borg 6–20 scale; (3) respiratory exchange ratio ≥ 1.10; and (4) a plateau in oxygen consumption in the final two stages of the test (defined as ≤150 mL of oxygen consumption difference between the last two stages of the exercise test). Participants also underwent maximal strength testing using standard protocols developed in our laboratory to determine their 1-repetition maximum in 6 resistance exercises (chest press, seated row, shoulder press, leg press, knee extension, and knee flexion).

### 4.2. Sample Size and Participants

A total of 6 participants (n = 4 male and n = 2 female) completed all aspects of this pilot study. We initially wanted to recruit 12 participants for this pilot project (6 males and 6 females) but the COVID-19 pandemic severely impacted recruitment. A total of 11 participants were initially recruited; however, 5 of these participants were lost to attrition due to lack of time (n = 2), travel plans that interfered with data collection (n = 1), and unknown reasons (n = 2). Due to the end of the funding for the study, recruitment was terminated following the 6 participants that were recruited for this research trial. The participants for this study consisted of young (18–30 years of age), healthy (no ‘yes’ answers on the Get Active Questionnaire, which screens for potential health issues precluding participation in exercise), and recreationally active individuals (≥150 min of moderate to vigorous physical activity per week for at least the past 3 months). Once participants were screened, they provided written informed consent and were enrolled in the study to begin laboratory testing (described above). Ethical approval for this study was obtained from the Research Ethics Board at the University of Manitoba (protocol: HE2022-0072).

### 4.3. Acute Exercise Sessions

There were three different types of exercise that were evaluated in this pilot research. These included (1) MICE, where participants cycled on an ergometer at 35% of the peak power output (POpeak) achieved during the $\dot{V}$O_2_peak test for 30 min [27]; (2) HIIE, where participants cycled on an ergometer at 15% of POpeak achieved during their $\dot{V}$O_2_peak test for 1 min and then at 90% of POpeak for 1 min, which was repeated for 10 sets in a row for a total exercise time of 20 min [27]; and (3) RE, where participants performed a whole-body resistance exercise routine (80% of 1-repetition maximum for 3 sets with 8 repetitions per set) using plate-loaded weight machines, including chest press, seated row, shoulder press, leg press, knee extension, and knee flexion. There was 1 min of rest between sets and 2 min of rest between exercises for the RE. The acute single bouts of exercise were completed in a randomized and counterbalanced method to achieve a balanced design.

### 4.4. Blood Sample Analysis

For each single bout of exercise described above, participants came to the lab and sat for 30 min of passive rest. Then, each participant had a baseline blood draw completed by a phlebotomist using venipuncture to collect ~6 mL of blood from an antecubital vein into a cooled EDTA-coated vacutainer blood draw tube. Each participant then underwent the exercise bout for that session. Once the exercise session was complete, each participant came back to the hematology lab to have an immediate blood (~3 min after exercise completion) draw taken using the same methodology as the first blood draw. Blood samples were collected again at the 1 and 3 h post-exercise time points in the same manner for a total of 4 blood draws per session. During the recovery period from the exercise, participants were allowed water ad libitum and allowed to ambulate to utilize the bathroom, if needed, but did not participate in any more moderate or vigorous physical activity/exercise. Once the blood was collected, it was processed in the same manner as reported in previous studies from our lab [28,29]. Briefly, blood-collection vacutainer EDTA tubes were centrifuged for 15 min at 3000 rpm at 4 °C to separate the plasma immediately after collecting it from the participants. Plasma was then aliquoted into 4 separate microtubes with approximately 500 μL of plasma in each microtube. The aliquots were immediately placed and stored in a −80 °C freezer. The plasma was stored at −80 °C until the assays were performed. For this study, we used multiplex technology to assay all 5 myokines in one assay using a commercially available kit (HMYOMAG-56K, MILLIPLEX MAP Human Myokine Magnetic Bead Panel, Oakville, ON, Canada). We completed the assays using Luminex xMAP Magpix^®^ multiplex technology (Luminex Corporation, Austin, TX, USA) according to standard operating procedures developed in our lab [28]. The intra-assay coefficient of variations ranged from 12.5 to 19.0% and the inter-assay coefficient of variations ranged between 2.9 and 16.7% for all the 5 myokines measured.

### 4.5. Statistics

Data was checked for normality using the Shapiro–Wilk test. All the myokine data was not normally distributed, so a logarithm transformation was completed to analyze the data. Further, the repeated-measures ANOVA data was checked for sphericity using Mauchly’s test of sphericity. If Mauchly’s test of sphericity was violated, a Greenhouse–Geiser correction factor was applied to the data. The results of the myokine data are reported in physiological concentrations to allow for a comparison with other work in this area of research. All myokines were evaluated using a 3 (exercise condition: MICE, HIIE, RE) × 4 (time: baseline, immediately post-exercise, 1 and 3 h post-exercise) repeated-measures analysis of variance with statistical significance set at *p* ≤ 0.05 using Statistica version 13.3 (Tibco Software, Palo Alto, CA, USA). For any significant interaction or main effects, a Tukey HSD post hoc comparison test was completed to determine where the differences were located. Effect sizes were calculated using partial eta squared (η_p_^2^) for all main and interaction effects. For the purposes of this study, small, medium, and large effect sizes were determined as 0.01, 0.06, and 0.14, respectively [30].

## Figures and Tables

**Table 1:** Baseline characteristics (mean ± SD) of individuals completing the three exercise protocols.

Variable	All (n = 6)	Males (n = 4)	Females (n = 2)	*t*-Test (*p*-Value) Males vs. Females
Age (years)	26.83 ± 4.17	26.00 ± 4.97	28.50 ± 2.12	0.55
Height (cm)	171.57 ± 5.25	171.98 ± 3.21	170.75 ± 10.25	0.82
Mass (kg)	70.23 ± 10.80	71.63 ± 2.94	67.45 ± 23.12	0.70
Fat-free mass (kg)	56.95 ± 9.49	60.00 ± 2.82	50.85 ± 17.75	0.32
Fat mass (kg)	13.28 ± 5.28	11.63 ± 5.08	16.60 ± 5.37	0.33

**Table 2:** Myokine response of three exercise types and all three types of exercise combined on apelin, FGF-21, IL-6, IL-15, and irisin.

Myokine	Exercise Type	Baseline	Immediately Post	1 h Post	3 h Post
Apelin (pg/mL)	MICE	298.60 ± 156.80	266.20 ± 97.09	271.95 ± 138.08	268.63 ± 131.07
	HIIE	268.27 ± 105.84	320.41 ± 147.33	281.17 ± 75.02	285.51 ± 84.21
	RE	279.27 ± 142.68	280.33 ± 87.14	234.65 ± 98.97	211.57 ± 112.12
	All	282.12 ± 129.15	288.98 ± 109.31	262.59 ± 102.86	255.24 ± 109.06
FGF-21 (pg/mL)	MICE	162.46 ± 147.27	184.58 ± 111.59	153.18 ± 153.52	113.35 ± 146.17
	HIIE	201.23 ± 119.39	217.85 ± 133.89	166.52 ± 144.20	139.70 ± 98.70
	RE	203.19 ± 133.86	216.38 ± 177.79	111.40 ± 144.53	80.20 ± 148.88
	All *	188.96 ± 127.34	206.27 ± 135.95	143.70 ± 140.62	111.08 ± 127.65
Irisin (pg/mL)	MICE	1033.59 ± 1322.80	633.34 ± 623.35	1326.79 ± 834.74	1557.02 ± 1196.32
	HIIE	586.43 ± 532.30	1838.01 ± 1752.78	1312.97 ± 719.21	1268.72 ± 1052.58
	RE	1421.29 ± 1531.44	1259.43 ± 1301.57	683.90 ± 709.60	741.71 ± 624.51
	All	1013.77 ± 1187.85	1243.59 ± 1301.57	1107.89 ± 774.84	1189.15 ± 991.04
IL-6 (pg/mL)	MICE	7.51 ± 8.17	6.26 ± 5.70	5.96 ± 8.23	7.61 ± 5.61
	HIIE	9.20 ± 6.39	8.89 ± 6.59	5.88 ± 6.11	6.60 ± 3.08
	RE	10.48 ± 6.88	8.35 ± 8.58	5.64 ± 7.01	7.49 ± 5.72
	All	9.06 ± 6.87	7.83 ± 6.73	5.83 ± 6.74	7.24 ± 4.68
IL-15 (pg/mL)	MICE	5.53 ± 4.26	4.35 ± 2.14	5.89 ± 3.66	6.12 ± 1.61
	HIIE	4.67 ± 3.20	5.73 ± 2.38	5.03 ± 2.62	4.88 ± 0.90
	RE	5.51 ± 2.99	5.36 ± 2.41	5.61 ± 2.41	4.41 ± 1.96
	All	5.24 ± 3.34	5.15 ± 2.46	5.51 ± 2.80	5.14 ± 1.64

FGF-21: fibroblast growth factor-21; HIIE: high-intensity interval exercise; IL-6: interleukin-6; IL-15: interleukin-15; MICE: moderate-intensity continuous exercise; RE: resistance exercise. * Significant main effect of time (*p* < 0.05); baseline and immediately post exercise significantly higher than 3 h post-exercise.

## Data Availability

The original contributions presented in this study are included in the article. Further inquiries can be directed to the corresponding author.
